# Patients’ Plasma Activity of Heparin, low-Molecular-Weight Heparin or no Anticoagulants on Urine Based DOAC Test Strips

**DOI:** 10.1177/10760296221083667

**Published:** 2022-03-11

**Authors:** Job Harenberg, Svetlana Hetjens, Christel Weiss

**Affiliations:** 1 DOASENSE GmbH, Heidelberg, Germany; 2 9144 Ruprecht-Karls University Heidelberg, Heidelberg, Germany; 3 Medical Faculty Mannheim, Ruprecht-Karls University Heidelberg, Heidelberg, Germany

**Keywords:** heparin, low-molecular weight heparin, anti-factor xa activity, point-of-care test, DOAC dipstick

## Abstract

DOAC Dipstick determines specifically the presence and absence of direct oral anticoagulants (DOACs) from patients’ urine samples and handmade test strips performed as well as the commercial version. To compare plasma activity (chromogenic substrate assays) from plasma samples with results from urine samples (DOAC test strips) of patients treated with heparin, low-molecular weight heparin (LMWH) and without anticoagulation. Plasma anti-factor Xa (aXa) activity was determined by Coamatic chromogenic substrate assay and compared to the presence of anticoagulants in urine by DOAC test strips. Patients were treated for least 5 days and samples were taken 4 hrs after administration in comparison to no treatment with an anticoagulant (n = 42). A total of 100 patients were included treated with heparin (n = 29), LMWH nadroparin (n = 29) or no anticoagulants (n = 42). Plasma aXa levels of patients treated with heparin (2 × 7.500 IU daily subcutaneously, 12 male, age 67.4 ± 11.5 years) were 0,18 IU/ml ± 0,15 IU/ml (mean, standard deviation), with LMWH (1 × 3000 IU daily subcutaneously, 15 male, age 64.2 ± 14.1 years) 0,17 IU/ml ± 0,16 IU/l, and with no anticoagulants (28 male, age 64.2 ± 15.6 years) 0,02 IU/ml ± 0.01 IU/ml. All factor Xa and thrombin inhibitor pad results of test strips were negative. We conclude that DOAC Dipstick has a high probability of not detecting heparin and LMWH in patients on treatment as well as in urine samples of patients not treated with an anticoagulant.

## Introduction

Direct oral anticoagulants (DOACs) may need to be detected quickly in patients’ emergency situation, which remains still a major challenge due to the lack of specific, sensitive methods with a short turn-around time.^1–3^ A number of coagulation tests are available in order to support a medical decision making in patients where DOACs need to be tested.^4–6^ A sensitive and rapid DOAC Dipstick test was developed to detect DOACs in urine samples.^7^ Direct factor Xa inhibitors (DXI) and thrombin inhibitors (DTI) are determined in one test only and colors of pads of DOAC Dipstick can be identified by naked eye after 10 min.^8^ The accuracy of the CE (Conformité Européenne) -labelled DOAC Dipstick test in detecting oral apixaban, edoxaban, rivaroxaban, and dabigatran in urine samples was described in a large multicenter trial.^9^ A meta-analysis of prototype and commercial type of test strips for detection of DOACs in urine showed identical performances reinforcing the confidence in DOAC Dipstick test.^10^

Factor Xa specific chromogenic substrate assays detect accurately heparin, LMWH and fondaparinux and thrombin specific chromogenic substrate assays to detect direct thrombin inhibitors from plasma samples of patients.^6^ DXIs and DTI dabigatran are also detected by these assays in plasma.^1,2^ Urine samples detect DOACs by DOAC Dipstick^5^ but when spiked with heparin or LMWH did not detect heparin.^11^ In the present investigation, we compared plasma anti-factor Xa levels with results of factor Xa and thrombin inhibitor test pads of a urine test strip from urine samples of consecutively to hospital admitted patients treated with heparin, LMWH nadroparin and with no anticoagulant treatment.

## Subjects and Methods

Hospitalized patients were included into the aged 18 years and older, and after having given written informed consent. The investigation was approved by the local ethics committee according to the Declaration of Helsinki 1964. It was conducted in a non-randomized fashion by including participants consecutively between September and December 2014. Exclusion criteria regarded patient who were unable to understand the content of the informed consent form. Plasma and urine samples of patients were analyzed at day 5 of anticoagulation with heparin (7.500 IU bid subcutaneously), LMWH nadroparin (3.000 IU od subcutaneously) or before anticoagulation. During the conduction of the investigation, urine and plasma samples of patients on treatment with DOACs were investigated in parallel by DOAC Dipstick and chromogenic substrate assays and were not reported as positive control group,^12,13^

### Preparation and Analysis of Plasma and Urine Samples

Blood samples (10 ml) were taken 4 hrs after subcutaneous administration of heparin or LMWH by venipuncture and was collected into plastic vials with sodium citrate (3.8%; blood/citrate 9/1 v/v), centrifuged at 1800 g for 10 min at room temperature within 2 h. Platelet poor plasma was shock frozen in liquid nitrogen and stored at –80 °C for analysis within 8 weeks. Coamatic chromogenic substrate assay was used to determine the anti-factor Xa activity from plasma samples.^14^ All assays were run on the microtiter plate system Multiskan FC connected to the software program SkanIt 3.1 (Thermo Fisher Scientific, Langenselbold, Germany). Standard curves were computed using serial concentrations of heparin and nadroparin ranging from 0.01 to 1.0 aXa IU added to normal human plasma pooled from 20 volunteers.^15^

Urine samples were collected immediately after blood sampling in 100-ml propylene containers with integrated units for closed transfer of urine to a V-urine vacuum system (Saarstedt, Nuermbrecht, Germany). Urine samples (4 ml) were transferred into polyethylene terephthalate tubes (V-Monovette urine Z4 ml, Sarstedt AG, Nuermbrecht, Germany) using plastic syringes and analyzed within 10 min and analysed within 2 h by urine test strips.

Test strips contained reagents to detect factor Xa and thrombin inhibitors were immersed in urine samples for 2 to 3 s and processed as described previously.^8^ Test strips are left undisturbed for 10 min to allow the chemical reaction to take place on the pads. Test pads show specific colors depending on the presence or absence of factor Xa inhibitor or thrombin inhibitor anticoagulants that can be identified by naked eye after 10 min: colors white and rose in presence and yellow and ochre in absence of the inhibitors, respectively. Two trained observers, who were blinded to the subjects’ treatment, adjudicated independently the colors of the test pads and in case of disagreement a third examined person evaluated the pads of the test strips.

## Statistics

All statistical calculations were performed using SAS software, release 9.4 (SAS Institute Inc., Cary, NC, USA). The qualitative data were presented as absolute and relative frequencies. The quantitative data were presented as mean and standard deviation (SD) and tested by 1-way ANOVA test or chi-squared test as applicable. The association of the Coamatic test with the evaluation of factor Xa and thrombin inhibitor pads was investigated and if required, tested by Chi-square of Fishers exact test at a level p < 0.05.

## Results

Of a total of 100 patients, 29 each were treated with heparin and LMWH and 42 were not treated with an anticoagulant which was before anticoagulant therapy. The biographic data of patients were not different regarding sex, age, weight, and body mass index between groups treated with heparin, LMWH without anticoagulant treatment (Table 1, p-values > 0.05). All patients presented a mean number of 7.7 diagnoses across all groups. The differences of frequencies of the individual diagnoses (p < 0.0005) were regarded as clinically not relevant mostly due to multiple diagnoses per patient defining them as multimorbid.

**Table 1.** Biographic Data of Patients.

	Heparin	LMWH	No Anticoagulant	*P*-value
N	29	29	42	
Male / female (n/n)	12/17	15/14	28/14	0.0998
Age, years (mean, SD),	70.0 (9.8)	62.9 (14.2)	63.6 (16.3)	0.1008
Bodyweight, kg (mean, SD)	80.2 (21.3)	79.0 (21.2)	76.4 (19.9)	0.7393
BMI (mean, SD)	28.5 (7.5)	27.3 (7.4)	26.5 (5.5)	0.4638
Heparin dose/day, IU (mean)	15000		-	n.a
LMWH dose/day, IU (mean)		3000	-	n.a
Diagnoses				
Cardiopulmonary disease	95	84	79	0.0005
Bronchopulmonary disease	32	18	40	
Metabolism disease	41	55	51	
Gastrointestinal disease	16	35	24	
Malignant disease	16	24	36	
other	29	37	58	

The values for aXa inhibition were 0.18 IU/ml ± 0.15 IU/ml, 0.17 IU/ml ± 0.16 IU/ml plasma, and 0.02 IU/ml ± 0.01 IU/ml for patients treated with heparin, LMWH and no anticoagulant. The factor Xa inhibitor and thrombin inhibitor pads of test strips were all negative in all 3 groups of patients, ie during treatment with heparin, LMWH and no anticoagulant therapy, respectively (Table 2). There were no discrepancies of the color by the adjudication of pads of the test strips by the two observers.

**Table 2.** Results of Anti-factor Xa Activity (aXa) in Plasma and of Factor Xa (FXA) and Thrombin Inhibitor (THR) Pad of DOAC Test Ttrips in Urine from Patients Treated with Heparin, LMWH and No Anticoagulant therapy.

	AXa Activity Plasma aXa IU/ml Mean / SD	FXA Inhibitor Pad of Urine Test Strip Negative / All n/n	THR Inhibitor Pad of urine Test Strip Negative / Alln/n
Heparin, n = 29	0,18 ± 0,15	29/29	29/29
LMWH, n = 29	0,17 ± 0,16	29/29	29/29
No anticoagulant, n = 42	0,02 ± 0,01	42/42	42/42

## Discussion

In this investigation, we have shown that test strips for detection of direct oral factor Xa and thrombin inhibitors does not detect heparin and LMWH in urine samples of patients. The amount of active heparin in urine is about 30%^16^ and the test strip may react with immobilized factor Xa and thrombin specific reagents on the pads. The findings may be explained by the absence of antithrombin or heparin cofactor II in normal urine,^17,18^ which are required for binding to and inhibition of the coagulation enzymes by heparin and LMWH.

The reagents that are immobilized onto test strips pads specifically detect direct factor Xa or thrombin inhibitors and do not interact with one another.^8^ We have reported that urine samples, spiked with the indirect factor Xa and thrombin inhibitors heparin and the predominantly factor Xa inhibiting low molecular weight heparin do not react with the pads of the test strip. This contrasts with coagulation tests using blood and plasma samples, which detect specifically heparin and LMWH at low concentrations,^19^ Therefore, the lack of interaction of heparin and LMWH with the test strip pads could not be deduced with certainty from urine samples of patients treated with these anticoagulants.

The present data have shown that urine of patients who did not receive an anticoagulant did not react with the immobilized reagents of the test strips and gave therefore a negative result. This supports earlier reported findings in a smaller patient group without anticoagulant therapy.^11^ In addition, results of pads of the test strip did not show interactions by coumadin treated patients.^11^

There are some limitations to this study. A pre-determined hypothesis and an associated sample size calculation were not performed and data may be regarded as of observational nature only and would require further confirmation utilizing an appropriately designed trial approach. However, the results of our investigation may be regarded as confirmatory following results from intro vitro experiments^11^ but still may require confirmation. Patients were not included if treated with fondaparinux, dalteparin, hirudin and argatroban because they were not available during the study period. In vitro studies did not show an interaction of fondaparinux with the test pads of test strips and the lack of interaction of LMWH up to 1 IU/mL plasma, which in the present study makes it highly unlikely that this may be different from urine samples of patients treated with fondaparinux as well as with danaparoid. However, treatment with higher doses of heparins remain to be investigated to confirm the lack of interaction with DOAC Dipstick. Some interaction of r-hirudin and argatroban was reported by in vitro studies^11^ but they were not available during conduction of the study. The use of urine test strips may not add a benefit for these patients because dose adjustments are performed with specific clotting assays.^20^ We used the prototype version of test strips for this study. However, a meta-analysis of the results obtained with the prototype of test strips versus the commercial DOAC Dipstick type did not identify differences in the accuracy.^10^

In summary, the present ex vivo investigation confirms in vitro data,^11^ that DOAC Dipstick has a high probability of not detecting heparin, LMWH and urine samples of patients not treated with an anticoagulant. These data substantiate the specificity of the test system and differs from that of blood or plasma-based test systems.

SH, CW do not have to declare conflicts of interest.
